# Research advances in the clinical application of esketamine

**DOI:** 10.1002/ibra.12019

**Published:** 2022-03-05

**Authors:** Xiao‐Xi Zhang, Nai‐Xin Zhang, De‐Xing Liu, Jun Ding, Yi‐Nan Zhang, Zhao‐Qiong Zhu

**Affiliations:** ^1^ Department of Anesthesiology Affiliated Hospital of Zunyi Medical University Zunyi Guizhou China

**Keywords:** analgesia, anesthesia, conscious sedation anesthesia, esketamine, sedation

## Abstract

Esketamine is dextrorotatory ketamine, which is an enantiomer of ketamine. Compared with ketamine, it has the advantages of a fast metabolism, fewer side effects, and strong pharmacological effects, so it is more suitable for clinical use. Esketamine has a powerful analgesic effect and has little effect on breathing. It has a wide range of applications in the fields of pediatric anesthesia, conscious sedation anesthesia, and emergency analgesia. In addition, it is also used for pain that is difficult to relieve with conventional drugs and to prevent postoperative pain. Various routes of administration are also suitable for patients who need short‐term analgesia and sedation. As a drug, esketamine inevitably brings some side effects when it is used clinically. In this article, by introducing the mechanism of action and pharmacological characteristics of esketamine, its clinical application is reviewed, and it provides a reference for the more reasonable and safe clinical application of esketamine.

## Graphical abstract

This article describes esketamine from four aspects, namely the mechanism, common clinical applications, common side effects, and prospects for esketamine. It describes the advantages of esketamine at the current stage, its unique role in some special fields, and compares it with ketamine.

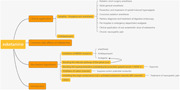

## 1 INTRODUCTION

R‐Ketamine and S‐Ketamine are optical enantiomers of the ketamine. Esketamine, which was recently approved for the treatment of depression, is essentially S‐ketamine. Esketamine is approved by the U.S. Food and Drug Administration for the treatment of depression in which two or more antidepressants are ineffective. The nasal administration of esketamine spray can significantly improve the symptoms of depression patients who are at risk of suicide. Depressive symptoms have a long‐lasting effect and delay recurrence, so it has attracted the attention of researchers.^1^, ^2^ Ketamine has a powerful sedative and analgesic effect and has been used in clinical anesthesia for more than 50 years.^3^ However, the side effects of ketamine are obvious, such as increased blood pressure and intracranial pressure, ascension of airway secretions and heart rate, dizziness, vomiting, hallucinations, and so forth, making it gradually replaced by other anesthetics. The pharmacological characteristics of S‐ketamine are similar to ketamine. Studies have shown that the affinity of S‐ketamine for *N*‐methyl‐d‐aspartate (NMDA) receptors is about three times that of ketamine. Therefore, its analgesic and anesthetic effects are about two to three times that of ketamine.^3^, ^4^, ^5^, ^6^ Compared with ketamine, S‐ketamine is metabolized faster in the body and has minor side effects. The dose of S‐ketamine used to induce anesthesia is half that of ketamine, and the resuscitation time is one‐third that of ketamine.^6^, ^7^, ^8^, ^9^

## 2 THE MECHANISM OF ACTION OF ESKETAMINE

**Figure 1:**
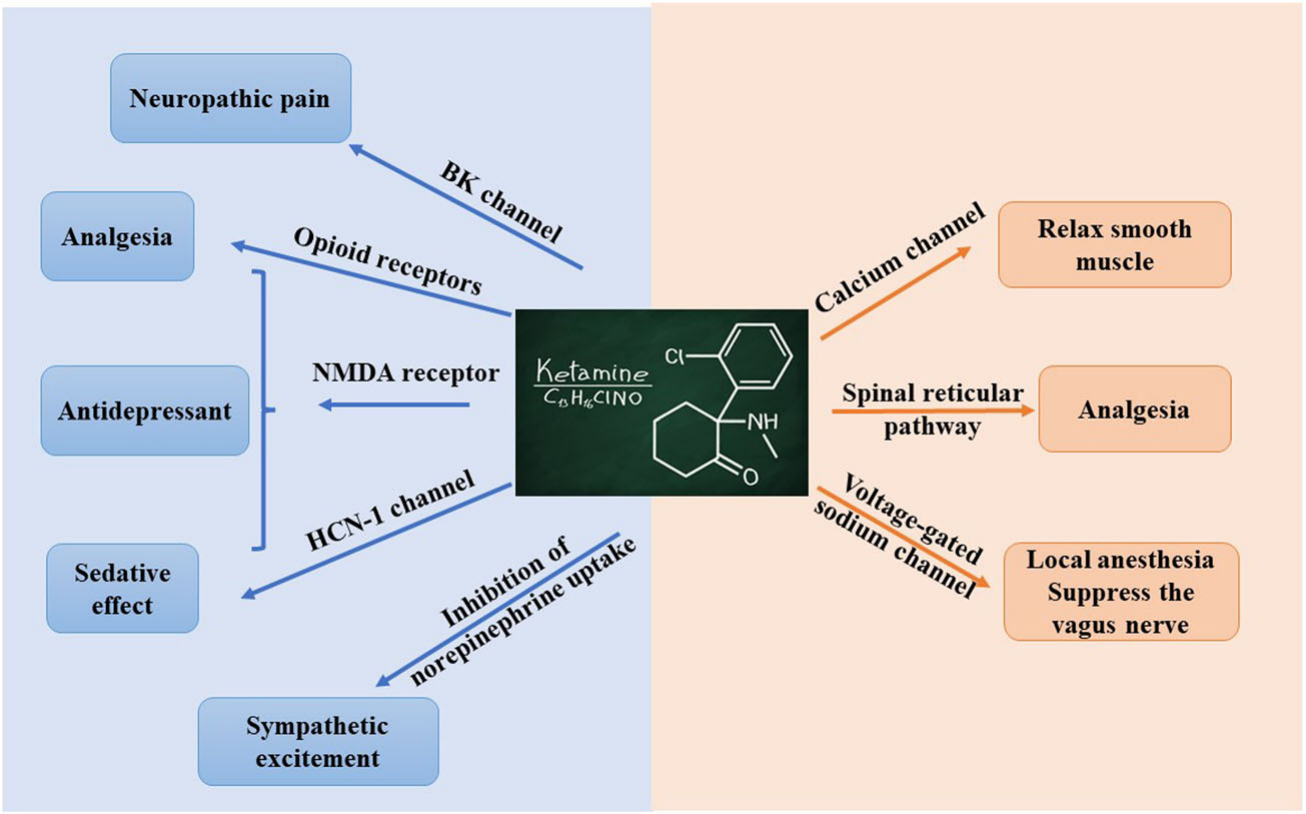
An overview of mechanism hypothesis: the action of esketamine. The blue part on the left side of the figure is the mechanism hypothesis and clinical action characteristics of esketamine supported by the literature. The orange part on the right is based on the mechanism of ketamine and the clinical effect of esketamine. The mechanism hypothesis has not been reviewed in recent years to support it [Color figure can be viewed at wileyonlinelibrary.com]

Current research suggests that ketamine acts on multiple receptors and channels causing different effects. The mechanism of action of S‐ketamine is similar to that of ketamine. There are several hypotheses regarding the mechanism of action of S‐ketamine:

- S‐ketamine inhibits NMDA receptors and exerts anesthesia, amnesia, analgesia, anti‐hyperalgesia, antidepression and other effects. When neuronal cells are in a resting state, Mg^2+^ binds to NMDA receptors to prevent Ca^2+^ from entering cells. When glutamate and glycine bind to NMDA receptors at the same time, NMDA receptors are activated and releases Mg^2+^, and a large amount of Ca^2+^ enters neuronal cells, activates secondary messengers, and produces NO, prostaglandins, and other substances. Excessive production of NO can directly inhibit GABA receptor activity, which reduces presynaptic inhibition. NO can also promote the release of presynaptic glutamate and other ways to participate in the regulation of nociception^10^, ^11^, ^12^, ^13^, ^14^; Prostaglandins can also amplify the conduction of pain signals.^12^, ^15^ Both ketamine and S‐ketamine inhibit the activation of downstream signaling pathways by inhibiting NMDA receptors and exerting analgesic effects.^6^, ^16^, ^17^, ^18^
- Blocking the spinal cord reticular pathway at the level of the spinal cord: The sensory input generated by nociceptors activates the secondary neurons in the spinal cord, and the secondary neurons project to the somatosensory cortex through the intermediate relay area (including the thalamus), which ultimately triggers pain perception. Ketamine blocks the incoming pain signals from the spinal cord reticular pathway and exerts analgesic effects.^3^, ^19^
- Blocking the hyperpolarization‐activated cyclic nucleotide‐gated channel‐1 (HCN‐1) produce a hypnotic effect (HCN‐1 is mainly expressed in the cortex and affects the excitability of neurons by changing cell resting potential and membrane resistance).^20^, ^21^, ^22^, ^23^
- Inhibition of cation channels: Blocking voltage‐gated sodium channels exertlocal anesthesia; restrainng potassium channels in spinal dorsal horn neurons can reduces neuronal excitability^24^; blocking L‐type Ca^2+^ channel can relax smooth muscle^6^; blocking sodium channels of the brainstem parasympathetic can diminish cardiac parasympathetic nerve activity that raises heart rate and blood pressure.^25^ This effect is also related to S‐ketamine inhibiting the uptake of norepinephrine by neurons.^9^
- Inhibiting the large‐conductance Ca^2+^‐activated potassium channels (BK channels) can treat neuropathic pain: The excessive activation of spinal cord microglia after peripheral nerve injury is involved in the development of neuropathic pain. S‐ketamine inhibits the excessive activation of microglia by blocking BK channels to relieve neuropathic pain.^26^
- S‐Ketamine also acts on opioid receptors, and its affinity for opioid receptors is about twice that of ketamine, but opioid receptor antagonists cannot reverse the analgesic effect of ketamine, indicating that opioid receptors may not participate in ketamine's analgesic effect^27^, ^28^ (Figure 1).

However, different channels are not isolated, but are part of a complete nervous system, with countless interactions at all levels. This is obvious in terms of antidepressant: molecular design based solely on NMDA receptor inhibition is not success that because pure NMDA receptor antagonists cannot show rapid and sustained antidepressant effect.^17^ Because esketamine acts on a variety of receptors and channels, it is difficult to avoid some side effects in clinical use, and these side effects limit its use to a certain extent. Common side effects of the esketamine are increased heart rate and blood pressure, nausea or vomiting, impaired vision, dizziness, and psychomotor agitation,^3^ which require clinicians to carefully evaluate when using the drug to reduce the risk.

## 3 CLINICAL APPLICATION OF ESKETAMINE

### 3.1 Treatment‐Resistant depression

After esketamine was approved by the U.S. Food and Drug Administration for the treatment of refractory depression, there have been many clinical studies on its efficacy. Subcutaneous injection (0.5–1 mg/kg) of esketamine, once a week for six weeks, can improve anhedonia symptoms in patients with unipolar and bipolar depression.^29^ For patients with major depression who have active suicidal ideation, compared with the specific delayed onset characteristics of traditional oral antidepressants, the use of esketamine nasal spray can quickly and effectively relieve the symptoms of depression.^30^ Patients over 65 years of age with refractory depression were randomly given three different doses of esketamine (28, 56, 84 mg) nasal sprays twice a week for four weeks. There was no significant improvement in depression symptoms at the end of treatment.^31^ Studies have also shown that patients aged 18–64 have continued treatment for four weeks (twice a week, at a dose of 56 or 84 mg), and the symptoms of patients who did not respond early in the treatment period improved significantly after the treatment cycle.^32^ In another clinical study, when esketamine was used in two groups of 18–64 years and older than 65 years, young patients and elderly patients had the same degree of improvement in depressive symptoms and the safety of each age group was generally consistent. No new safety issues have been discovered in both age group.^33^ There are many side effects when using esketamine to treat depression, such as increased blood pressure, dizziness, and nausea. For adults who inhale esketamine (dose range of 28–84 mg) through nasal spray once or twice a week to treat depression (treatment duration is 4–52 weeks), the drug will bring a short‐term increase in blood pressure. Blood pressure generally reaches the highest value 40 min after administration and drops to the pre‐administration level about 1.5 h. When used in combination with oral antidepressants, there is no short‐term or long‐term effect on olfactory function.^35^ The use of this dose (<84 mg) will not damage the patient's cognitive function.^36^, ^37^ and there is no obvious urinary tract toxicity in the short‐term use.^38^ After intranasal administration, the absolute bioavailability of esketamine at the usual doses (28, 56, and 84 mg) is >50%, the plasma concentration is linearly eliminated, and there is no accumulation when administered twice a week.^39^ Some researchers have found that a single intravenous injection of esketamine (0.25 mg/kg) and ketamine (0.5 mg/kg) has a similar relieving effect on refractory depression after 24 h, and there is no significant difference in side effects, but the effect of ketamine is longer.^40^ This makes clinicians question the advantages of esketamine in the treatment of depression. Although the mechanism of action of esketamine in treating depression is not clear, and side effects are common, the current clinical evidence is biased towards esketamine can alleviate the depressive symptoms of adults with refractory depression.^41^ A risk‐benefit assessment also confirmed that the benefits of esketamine in the treatment of refractory depression outweigh the adverse effects it brings.^42^ In short, the current clinical trial results of esketamine in the treatment of depression are very different, and there is no authoritative guideline. Part of the reason is that the test conditions are complicated and the standards are not uniform. Secondly, the mechanism of action is complex, involving multiple signal transduction pathways, and its main mechanism in the treatment of depression has not yet been clarified. For better efficacy and rational use of drugs, a large number of studies are needed to confirm the true efficacy and mechanism of action of esketamine on patients with depression.

### 3.2 Pediatric short surgery anesthesia

Ketamine has many advantages in clinical pediatrics. It retains upper respiratory tract muscle tone and protective airway reflex. The common adverse reactions for ketamine in adults are less common and milder in children and adolescents.^43^ Adults do not need sedation to cooperate with doctors to complete the diagnosis and treatment of many diseases, while children need sedation and analgesia. For example, propofol combined with ketamine is a commonly used sedation technique when children undergo MRI examination, which has the characteristics of fast induction and quick recovery.^44^ The combination of midazolam and S‐ketamine can provide deep sedation for bone marrow aspiration.^45^ In pediatric wound care, rectal administration of ketamine and midazolam can produce effective sedation and analgesia without the need for additional drugs, but the use of higher doses of ketamine will prolong the recovery time.^46^ Compared with ketamine, esketamine has a stronger analgesic effect. Theoretically, the same analgesic effect can be obtained with a lower dose, suggesting that rectal administration of esketamine may provide a new way for pediatrics to provide short‐term sedation and analgesia. Children with acute intussusception are often reset by air or water pressure enema. Both air and water pressure enema require good analgesia and sedation. In a retrospective case cohort comparison study, morphine and esketamine were compared (*n* = 37 in the morphine group, *n* = 20 in the esketamine group). The results show that esketamine can improve the success rate of reduction, and shows a trend of reducing recurrence, shortening the time of operation and hospital stay. A small number of patients experienced vomiting events, which did not cause serious adverse events and was not statistically significant. (The dose of esketamine is 0.5–1 mg/kg, moderate sedation.)^47^ Esketamine has a powerful analgesic effect, fast onset, and fast metabolism, making it suitable for pediatric short surgery anesthesia.

### 3.3 Adult general anesthesia

At present, esketamine has been used in adult anesthesia. After sevoflurane is used to induce anesthesia, a laryngeal mask is inserted, and esketamine 0.5 mg/kg (or 1 mg/kg) is injected intravenously. After that, the dose of 0.5 mg/kg/h (or 1 mg/kg/h) was given by continuous intravenous infusion. Studies have confirmed that esketamine reduces the minimum alveolar concentration of sevoflurane in a dose‐dependent manner, indicating that esketamine has a coordinated effect with volatile anesthetics. However, patients taking esketamine 1 mg/kg have postoperative nausea.^48^ Further research on the effect of esketamine on sevoflurane will help anesthesiologists fully understand the interaction between the two, which is of great significance to clinical practice. Compared with propofol combined with sufentanil for induction of anesthesia in elderly surgical patients, propofol combined with esketamnie (0.5 mg/kg) is more stable in hemodynamics, but also can improve surgical stress and inflammation, and shorten anesthesia recovery time. There is no difference in the incidence of adverse reactions between the use of this low‐dose esketamine and the use of sufentanil.^49^ Some scholars believe that when anaesthetizing patients with severe depression, it may be beneficial to give preference to esketamine, but it lacks clinical evidence and needs to be confirmed.^50^ For some specific operations, such as breast disease surgery and laparoscopic hysterectomy, it will bring great psychological pressure to patients. S‐ketamine administered after induction of anesthesia can improve the mood of patients in the short term after surgery, and has an analgesic effect.^51^, ^52^ It shows that esketamine, as an auxiliary drug for anesthesia, is beneficial to the recovery of patients after surgery.

### 3.4 Prevention and treatment of opioid‐induced hyperalgesia

Patients who use opioids to control pain may be more sensitive to pain. Unlike drug resistance and addiction, it is another side effect of opioid therapy, called hyperalgesia caused by opioids.^53^ The mechanisms by which opioids induce hyperalgesia are complex and diverse, and the activation of NMDA receptors plays a key role in its development.^54^ It has been confirmed in animal experiments that long‐term opioid treatment can cause increased presynaptic NMDA receptor activity, which will promote the development of opioid‐induced hyperalgesia and opioid tolerance. Blocking NMDA receptors at the spinal cord level completely blocks hyperalgesia, but only partially weakens the analgesic tolerance produced by opioids.^55^ In clinical trials, in patients undergoing major abdominal surgery, intraoperative administration of relatively large doses of remifentanil increased postoperative pain sensitivity, especially hyperalgesia around the incision, while intraoperative use of small doses of ketamine can prevent opioid‐induced hyperalgesia.^56^, ^57^ Some patients with cancer whose pain symptoms are not relieved by opioids receive continuous low doses of ketamine, which reduces pain and the need for analgesics.^58^ At present, the effect of esketamine in preventing and treating opioid‐induced hyperalgesia is not very clear, and there are relatively few studies in this area. Most clinicians have insufficient knowledge of opioid‐induced hyperalgesia and lack an authoritative guide. Esketamine has a stronger affinity for NMDA receptors than ketamine, and theoretically, it should have a better effect. Esketamine can be tried in some patients who require long‐term opioid pain relief.

### 3.5 Conscious sedation anesthesia

In some specific operations, compared with general anesthesia, conscious sedation anesthesia can avoid tracheal intubation, reduce the physiological interference to the patient, and be more conducive to some specific surgical operations and patient recovery. High‐intensity focused ultrasound ablation (HIFU) is a therapeutic modality that induces thermal necrosis of biological tissues by focusing high‐energy ultrasound waves at one specific target.^59^ It has been used clinically. Studies have shown that propofol combined with esketamine (0.2 mg/kg/h) for sedation and analgesia can reduce the discomfort and pain during MRI‐guided HIFU treatment to an acceptable level. This sedation technology can also improve the stability of cardiovascular and breathing, and make it easy to synchronize the breathing pattern with the magnetic resonance HIFU signal, so as to provide interventional radiologists with the best treatment conditions, which can replace general anesthesia.^60^ In acute ischemic stroke (AIS) patients, propofol combined with esketamine for conscious sedation is safe and feasible, while reducing the use of opioids and maintaining the relative stability of hemodynamics.^61^ In a study investigating the effects of anesthesia on the prognosis of patients undergoing mechanical thrombectomy for AIS, it was confirmed that conscious sedation anesthesia is better than general anesthesia.^62^ It shows that esketamine combined with propofol can provide conscious sedation anesthesia, and this sedation technique can replace general anesthesia in some specific operations. The dose of esketamine used under conscious sedation anesthesia is less, the incidence of adverse reactions is low, and a small number of patients may suffer from drowsiness and dizziness.^60^

### 3.6 Painless diagnosis and treatment of digestive endoscopy

Endoscopy has been widely used in the diagnosis and treatment of gastrointestinal diseases. Due to limited medical resources, anesthesiologists must consider the safety and comfort of the patient as well as the recovery time. Therefore, the use of analgesic and sedative drugs must be reasonable and precise. Recent studies have confirmed that a single dose of esketamine/ketamine combined with propofol for painless gastroscopy is generally safe and tolerable. The patients were randomized to receive esketamine 0.5 mg/kg or ketamine 1 mg/kg while the propofol administration method and relative dose are the same for all patients. Both methods can provide satisfactory analgesia. The esketamine group had a shorter recovery time, and the incidence of adverse events, such as dizziness, restlessness, nausea, vomiting, headache, fatigue, and so forth, was also lower than that of the ketamine group.^63^ In the elderly, esketamine (0.5 mg/kg) combined with propofol is equally safe and effective for gastroscopy, and hemodynamics is more stable than propofol alone.^64^ When propofol is used for sedation, it can produce different degrees of respiratory depression.^65^ Therefore, the dosage of propofol should be minimized while ensuring adequate sedation. Compared with low‐dose esketamine combined with propofol and alfentanil combined with propofol sedation, low‐dose esketamine clearly reduces the total amount of propofol required for sedation during endoscopic retrograde cholangiopancreatography. There were no significant differences in recovery time, satisfaction of patients and endoscopists, and occurrence of side effects, such as respiratory or cardiovascular adverse events.^66^ In this study, esketamine reduced the dosage of propofol, and the incidence of adverse events was not different. The reason may be that the total dosage of propofol after the combined administration did not reach the blood concentration that could obviously cause respiratory depression. Studies have confirmed that esketamine reverses the respiratory depression induced by remifentanil in a dose‐dependent manner.^67^ It indicates that esketamine may have an antagonistic effect on respiratory depression caused by opioids. It has been reported that bariatric surgery patients with sleep‐disordered breathing were anesthetized with ketamine combined with propofol. No opioids were used during the perioperative period. The patient wakes up quickly without hypoxia, airway obstruction, or apnea. This method also has a certain postoperative analgesic effect.^68^ Esketamine is used as an auxiliary analgesic, combined with opioids and propofol for digestive endoscopy diagnosis and treatment, to reduce the dosage of opioids and propofol, or to replace opioids. For there is respiratory insufficiency itself or contraindicated in patients with opioid use, this may be a reasonable option.

### 3.7 Pre‐hospital or emergency department analgesia

Studies have confirmed that the use of esketamine in emergency situations is reliable and effective. In pre‐hospital emergency operations, patients often need analgesia, and sometimes it is difficult to open vascular access, and the way of administration is limited. Instillation of esketamine into the nasal cavity (maximum dose of 1 mg/kg for adults, 1.5 mg/kg for children) can effectively control pain, and the administration is convenient and fast. Under the condition of providing satisfactory analgesia, only a few patients have dizziness, and other adverse reactions are relatively rare.^69^, ^70^ In a prospective observational study, the use of esketamine (4 mg/kg) nebulized inhalation for children aged 4 months to 16 years in the emergency department can quickly relieve pain, and the simultaneous use of midazolam can achieve sedation and analgesia.^71^

### 3.8 Clinical application of sub‐anesthetic dose of esketamine

There are often different degrees of pain after surgery. Severe postoperative pain will affect the patient's recovery. It is related to the treatment result and brings more pain to the patient. Therefore, controlling postoperative pain is also a key part of the treatment process and cannot be ignored. Intraoperative opioid analgesia is the core part of general anesthesia. Intravenous or oral opioids are also one of the most common postoperative analgesia, clinically.^72^ However, opioids have a variety of acute side effects, including nausea, pruritus, respiratory depression, and constipation.^73^ These side effects not only lead to prolonged hospital stay but also lead to addiction, hyperalgesia, and chronic pain.^74^ With the deepening of the understanding of opioids, more and more scholars have proposed “opioid‐free anesthesia”.^75^ There is a trend to reduce the use of opioids during the perioperative period, such as increasing non‐opioid adjuvant drugs, using nerve block techniques, and so forth. Sub‐anesthetic dose of S‐ketamine as an auxiliary drug for general anesthesia can effectively assist analgesia and reduce the intensity of pain and the need for opioids in a short period of time after surgery.^76^ For patients undergoing laparoscopic cholecystectomy under general anesthesia, S‐ketamine (0.3 mg/kg/h) intravenous continuous infusion after induction of anesthesia until the end of the operation is stopped, which can effectively control postoperative pain and reduce forthputting postoperative analgesic drugs.^77^ In another study, patients undergoing elective major abdominal surgery, a low‐dose S‐ketamine (0.015 mg/kg/h) continuous intravenous infusion for 48 h after induction of anesthesia can significantly reduce the hyperalgesia of the tissue around the incision and reduce postoperative opioid consumption, and ketamine‐related side effects are extremely rare.^78^ When patients with chronic opioid dependence undergo lumbar fusion surgery, S‐ketamine 0.5 mg/kg intravenously after induction of anesthesia, and continuous infusion at a rate of 0.25 mg/kg/h until the end of the operation, can significantly reduce the dosage of morphine within 24 h after surgery and has a tendency to reduce chronic pain six months after surgery, but the clinical effect is not accurate. The influence of intraoperative esketamine on postoperative chronic pain needs further research to confirm.^79^ PCA analgesia mode is used after lumbar fusion, and the addition of S‐ketamine can reduce the cumulative consumption of oxycodone within 24 h after surgery without additional side effects.^80^ However, some studies have confirmed that the use of S‐ketamine has no obvious advantage in postoperative pain. When non‐opioid dependent patients undergo lumbar fusion, intraoperative application of S‐ketamine does not reduce the need for postoperative analgesics (such as oxycodone) and has no significant effect on postoperative pain.^81^ After total knee arthroplasty, intra‐articular injection of S‐ketamine, although the visual analog score of postoperative pain decreased, was not statistically significant compared with the control group.^82^ The reason for the inconsistent results is not clear. Perhaps the time, method, and dose of administration will affect the final result. The effect of intraoperative ketamine on postoperative pain and consumption of analgesic drugs needs to be confirmed by a large number of studies (Table 1).

**Table 1:** Partial clinical study of esketamine in preventing and treating postoperative pain

Author and year	Sample size	Type of surgery	Method	Conclusion
Miziara, Simoni et al., 2016	Experimental group, *n* = 24 Control group, *n* = 24	Laparoscopic cholecystectomy	Experimental group: After induction of general anesthesia, 5 min before the start of the operation, S‐ketamine 0.3 mg/kg/h was continuously infused until the end of the operation Control group: infusion of the same amount of normal saline	The total consumption of opioids in the experimental group was low, and the visual analog pain score was low
Bornemann‐Cimenti, Wejbora et al., 2016	Low dose group, *n* = 18 Small dose group, *n* = 19 Control group, *n* = 19	Elective major abdominal surgery (colon, rectal and liver surgery)	Low‐dose group: After induction of anesthesia, intravenous injection of S‐ketamine 0.25 mg/kg, followed by continuous intravenous infusion of S‐ketamine 0.125 mg/kg/h for 48 h. Small dose group: After induction of anesthesia, an equal volume of normal saline was injected intravenously, followed by a continuous infusion of S‐ketamine 0.015 mg/kg/h for 48 h. Control group: infusion of the same amount of normal saline	Both experimental groups can reduce postoperative pain and reduce opioid consumption, and the incidence of postoperative delirium in the small‐dose group is lower than that of the low‐dose group
Nielsen, Fomsgaard et al., 2017	Experimental group, *n* = 74 Control group, *n* = 73	Lumbar fusion (using opioids every day for pain relief 3 months before the operation)	Experimental group: After induction of anesthesia, intravenous injection of S‐ketamine 0.5 mg/kg, and then S‐ketamine 0.25 mg/kg/continuous infusion until the end of the operation Control group: infusion of the same amount of normal saline	At 24 h after operation, there was no statistically significant difference in pain between the two groups. At 6 months after operation, the pain score of the experimental group was lower than that of the control group, but there was no statistically significant difference. Compared with preoperative pain, the experimental group had better pain improvement than the control group, which was statistically significant.
Brinck, Virtanen et al., 2021	Experimental group 1, *n* = 25 Experimental group 2, *n* = 25 Experimental group 3, *n* = 25 Control group, *n* = 25	Lumbar fusion	After the operation, the analgesic pump is connected to the intravenous channel, and the patient controls the medication according to the degree of pain. The drugs in the 4 groups of analgesic pumps contain oxycodone 1 mg/ml. Additional: Experimental group 1: +S‐ketamine 0.25 mg/ml; Experimental group 2: +S‐ketamine 0.5 mg/ml; Experimental group 3: +S‐ketamine 0.75 mg/ml	Within 24 h after surgery, the total consumption of oxycodone in the experimental group was significantly reduced
Maisniemi et al., 2021	Experimental group 1, *n* = 65 Experimental group 2, *n* = 62 Control group, *n* = 62	Lumbar fusion	Experimental group 1: S‐ketamine 0.5 mg/kg intravenous injection before operation, afterwards 0.12 mg/kg/h continuous infusion until the end of the operation. Experimental group 2: S‐ketamine 0.5 mg/kg intravenous injection before operation, afterwards 0.6 mg/kg/h continuous infusion until the end of the operation Control group: infusion of equal volume normal saline	There is no difference in the consumption of analgesic drugs between the two experimental groups and the control group within 48 h after surgery; at 4 h after surgery, the pain score of the experimental group is lower than that of the control group, which is statistically significant, and the pain relief is short‐lived
Guará Sobrinho, Garcia et al., 2012	Experimental group 1, *n* = 19 Experimental group 2, *n* = 17 Control group, *n* = 20	Total knee replacement	Experimental group 1: 0.25 mg/kg S‐ketamine dissolved in 20 ml saline. Experimental group 2: 0.5 mg/kg S‐ketamine dissolved in 20 ml saline Control group: 20 ml normal saline. All patients complete the operation under spinal anesthesia, before the surgical incision is completely closed, like an intra‐articular injection of medicine	At 2, 4, 6, 12, and 24 h after operation, the pain score of the experimental group was lower than that of the control group, but it was not statistically significant

### 3.9 Chronic neuropathic pain

According to the definition of the International Association for the Study of Pain, neuropathic pain can be caused by a variety of causes that affect the peripheral or central nervous system. Various pathologies or diseases involving the somatosensory nervous system will not only increase pain sensitivity but also cause spontaneous pain.^83^ The important mechanisms for the development of chronic neuropathic pain include the upregulation of NMDA receptors, the loss of downward inhibition, the plastic changes of the spinal cord caused by the release of pro‐inflammatory cytokines, and the activation of spinal cord immune cells.^84^ Ketamine, as a noncompetitive antagonist of NMDA receptors, has been used clinically to treat chronic neuropathic pain.^85^, ^86^ In addition, ketamine can adjust the downward inhibitory pathway to relieve pain,^87^ and even topical treatment of neuralgia after herpes zoster has a certain effect.^88^ However, some researchers have pointed out through MRI that the downward inhibitory pathway is not involved in the treatment of chronic neuropathic pain with ketamine.^89^ The therapeutic effect of ketamine on chronic neuropathic pain varies greatly. Recent studies have pointed out that long‐term intravenous infusion of low‐dose ketamine has no obvious alleviation effect on chronic neuropathic pain, and even if used in combination with magnesium sulfate, there is no significant improvement effect compared with placebo.^90^ There are relatively few studies on the use of S‐ketamine in the treatment of chronic neuropathic pain. In animal studies, S‐ketamine has been shown to treat neuropathic pain. The mechanism is to inhibit the large conductive potassium channels (BK channels) of the microglia in the spinal cord, thus preventing the overactivation of microglia. (Excessive activation of spinal cord microglia after peripheral nerve injury can lead to neuropathic pain).^26^ After receiving an intravenous infusion of 0.5 mg/kg of S‐ketamine, patients with fibromyalgia have a relief effect in a short period of time (45 min).^91^ Low‐dose S‐ketamine is continuously infused for 5 days, which can last a long time (12 weeks) to relieve pain in patients with type I complex regional pain syndrome.^92^ The clinical evidence of S‐ketamine in the treatment of chronic neuropathic pain is limited, its therapeutic effect is not clear, the mechanism is not clear, and a large number of clinical studies and observations are still needed.

## 4 COMMON SIDE EFFECTS OF ESKETAMINE

S‐ketamine can increase cerebral blood flow, causing increased intracranial pressure.^93^ But under normal circumstances, the increase in intracranial pressure caused by esketamine can be reduced when controlled ventilation or hyperventilation reduces the partial pressure of CO2 in arterial blood.^94^ In addition, it can also cause increased blood pressure and tachycardia.^95^ Firstly, esketamine can induce systemic release of catecholamines and inhibit the reuptake of norepinephrine by peripheral nerves and isoneuronal tissues such as cardiomyocytes. Second, esketamine can inhibit vagus nerve and make sympathetic nerve relatively excited. These factors can cause heart rate and blood pressure to rise.^96^ Ketamine's electrophysiological inhibition of thalamic cortical pathways and stimulation of the limbic system can cause dizziness, disturbance of consciousness, “separation state“ (The patient keeps his eyes open and corneal reflex and light reflex are present, accompanied by slow nystagmus), and other bad feelings when patients wake up from anesthesia. Long‐term use of ketamine can cause ulcerative cystitis, and short‐term use may also cause urinary incontinence.^97^, ^98^ This may be related to the ketamine antagonizing L‐type‐Ca^2+^ channel, inhibiting Ca^2+^ influx and urinary dysfunction caused by smooth muscle contraction.^99^ However, there is currently no study on the effect of long‐term use of esketamine on the bladder. S‐ketamine antagonizes muscarinic receptors, which may lead to increased upper respiratory tract secretions, resulting in obstructed airways in patients with general anesthesia.^100^, ^101^ However, esketamine is generally used as an auxiliary anesthetic, and there are no reports of serious respiratory adverse events. When esketamine is used as an antidepressant, side effects, such as separation, nausea, vomiting, anxiety, and increased blood pressure may also occur. Depressive symptoms may recur shortly after discontinuation of esketamine, and symptoms may worsen after the relapse. The incidence of such adverse reactions is greater in women than in men.^102^, ^103^ When used as an analgesic and sedative drug, the incidence of side effects of esketamine is lower than that of ketamine, the reaction is mild and tolerable, and there is no gender difference.^9^, ^11^ In short, there are no reports of serious adverse events caused by the clinical use of esketamine. Esketamine is used for short‐term analgesia and sedation, and there have been no reports of patients suffering from addiction.

## 5 PROSPECTS

Ketamine has been used clinically for many years. Due to its many side effects, it has been gradually replaced by other drugs. Recently, it was discovered that esketamine may have an antidepressant effect, and it has reappeared in the public eye. A large number of clinical studies are currently underway for its antidepressant effect, which hopefully will bring a new and effective treatment method for patients with depression. Esketamine has a stronger analgesic effect than ketamine, and has pharmacological effects such as anesthesia and sedation. Compared with opioid analgesics, esketamine has less effect on breathing that is an advantage in anesthesia where spontaneous breathing needs to be preserved. In terms of complex pain treatment and prevention of postoperative pain, the current clinical research results are quite different, and a large number of clinical studies are needed to confirm. The author believes that the focus of future research is still on the mechanism of action of esketamine and its effects in areas such as depression and complex pain. At present, there are also some urgent problems in these areas. To clarify the mechanism of action of esketamine can make more rational use of the drug, maximize its value, and better serve patients.

## CONFLICTS OF INTEREST

The authors declare that there are no conflicts of interest.

## ETHICS STATEMENT

The ethics statement is not available.

## AUTHOR CONTRIBUTIONS

Xiao‐Xi Zhang conceived and wrote the article under the guidance of Yi‐Nan Zhang, while Zhao‐Qiong Zhu, Nai‐Xin Zhang, De‐Xing Liu, and Jun‐Ding guided and assisted in the writing of the article and completed the final draft with the efforts of all authors. Zhao‐Qiong Zhu and Yi‐Nan Zhang is the corresponding author.

## TRANSPARENCY STATEMENT

All the authors affirm that this manuscript is an honest, accurate, and transparent account of the study being reported; that no important aspects of the study have been omitted; and that any discrepancies from the study as planned (and, if relevant, registered) have been explained.

## Data Availability

Data sharing not applicable to this article as no datasets were generated or analysed during the current study.
